# Characterization of Stable NiO*_x_*/SrTaO*_x_* N*_y_* Bilayers Boosting the Oxygen Evolution Reaction for Solar Water Splitting

**DOI:** 10.1002/smsc.202500638

**Published:** 2026-03-18

**Authors:** Zahra Pourmand Tehrani, Kyle J. Stephens, Vladimir Roddatis, Jochen Stahn, Aleksandar Staykov, Christof W. Schneider, Daniele Pergolesi, Thomas Lippert

**Affiliations:** ^1^ PSI Centre for Neutrons and Muons Sciences Paul Scherrer Institute Villigen Switzerland; ^2^ Laboratory of Inorganic Chemistry ETH Zürich Zurich Switzerland; ^3^ GFZ Helmholtz Centre for Geosciences Potsdam Germany; ^4^ International Institute for Carbon‐Neutral Energy Research (I^2^CNER) Kyushu University Fukuoka Japan; ^5^ PSI Centre for Energy and Environmental Sciences Paul Scherrer Institute Villigen Switzerland

**Keywords:** oxygen evolution reaction, oxynitride semiconductors, photoelectrochemical hydrogen generation, solar water splitting, solid–liquid interfaces

## Abstract

SrTaO_*x*_N_*y*_ (STON) is a well‐known visible light‐responsive semiconductor with ideally located band edges that allow the operability of overall water splitting. Like many oxynitrides, STON shows evidence of detrimental physicochemical changes under oxygen evolution reaction (OER) conditions involving strong caustic electrolytes. We investigate the development of STON instability with detailed electron microscopy and neutron reflectometry (NR) techniques using epitaxial thin films. Different crystallographic orientations are compared with ex situ analysis before and after OER in photoelectrochemical testing. A remarkable difference in stability of the STON surface is observed depending on the crystalline facets, with the [011] lattice planes being the more favorable orientation as compared to [001]. In addition, we show that the electrochemical stability of the photoelectrode surface can be dramatically improved by a homogeneous coating of NiO_*x*_, which significantly improves OER kinetics and surface stability in the alkaline environment. NR is realized in this work as a novel route to monitor this photoelectrochemical environment, and it lies in agreement with its microscopy counterpart to monitor the physicochemical changes. This demonstrates potential of NR as an alternative and complementary tool that also has the feasibility for future in situ experimental design.

## Graphical abstract

The oxynitride semiconductor SrTaO_*x*_N_*y*_ is a relevant photoactive material used for the hydrogen generation through solar water splitting. We report comparative results of structural, morphological, compositional, photoelectrochemical, and computational studies to elucidate the mechanisms leading to the electrochemical degradation during the oxygen evolution reaction of the bare semiconductor and the effect of NiO_*x*_ used as a protective/catalytic layer.

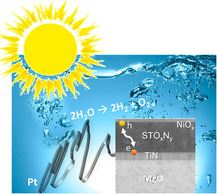

## 1 Introduction

Realizing a carbon‐neutral society requires cost‐effective and sustainable solar power capture procedures as well as storage methods. Among these methods is the energy conversion technology for solar fuel production, with hydrogen gas produced by water splitting being the main energy carrier [1]. Besides, H_2_ is not only a potential energy carrier (fuel), but it is also used for several very important chemical processes, such as the production of ammonia, which is among the main components for fertilizers.

The water splitting hydrogen production from renewable energy resources is nowadays based on two main routes, photovoltaic (PV) coupled with a water electrolyzer and direct photoelectrochemical (PEC) solar water splitting [2]. An estimate for the cost of the H_2_ produced using PV technology coupled with standard water electrolyzers is around €10 kg^−1^ H_2_ [3]. This is considerably larger than the price of H_2_ obtained via the steam reforming of fossil methane (ca. €1 kg^−1^) [4], although the enormous indirect geopolitical and environmental costs related to the use of fossil fuels are not taken into account for the cost evaluation.

Considering only renewable and carbon‐neutral routes for solar fuel production, PEC water splitting is a simpler and potentially cheaper concept compared to solar‐assisted PV‐electrolyzers [4, 5]. A techno‐economic analysis suggest, that PEC devices with reasonable manufacturing costs, a 10‐year lifetime, and a solar‐to‐hydrogen efficiency of 10% could produce hydrogen for a price as low as $7 kg^–1^ [5]. This evaluation puts (potentially) the cost of PEC‐based hydrogen production at a level similar to PV‐electrolyzers, and motivates our efforts to develop materials (or combination of materials) that can be processed as thin films with a higher solar‐to‐hydrogen conversion efficiency and a higher physicochemical stability under operating conditions. Further advances in this field require new physical insights into materials’ properties with respect to the photogeneration of charge carriers, charge transport, charge transfer at the solid/liquid interface, chemical stability, and (photo)catalytic activity.

The unavailability of electrochemically stable semiconductors that can effectively absorb photons in the visible spectrum and generate holes for the oxygen evolution reaction (OER) is one of the major obstacles that hinder the development of this concept [6]. The OER is, in fact, the main bottleneck of the water splitting process (2 H_2_O · 2 H_2_ + O_2_) because much higher overpotentials hamper this reaction compared to the hydrogen evolution reaction.

Many oxides have been investigated for this purpose over the last few decades. One major limitation of most oxide materials, however, is the wide band gap well above 3 eV. This allows only the utilization of photons in the UV energy range, which contribute to less than 5% of the solar spectrum.

The nitridation of oxide materials with an A_*x*_B_*y*_O_z_ structure to form AB(O, N)_3_ oxynitride perovskites is an effective approach to engineer semiconductors with a band gap in the visible. For this class of materials, the A cation can be, e.g., La, Sr, Ba, Y, or Ca, whereas the B cation can be occupied, among others, by Ti, Ta, or Nb. Moreover, for a number of oxynitride materials, the energy positions of the conduction and valence bands straddle the redox potentials for H^+^/H_2_ and O_2_/H_2_O, respectively [7]. These materials are therefore theoretically able to generate both H_2_ and O_2_ from water, a process often referred to as overall water splitting. With these two features fulfilled, several oxynitrides are classified as suitable photocatalytic materials for visible‐light‐driven solar water splitting at an irradiation wavelength of *λ* < 600 nm [8]. To achieve a better performance, the oxynitride surface is decorated with co‐catalysts [9], such as NiO, IrO_2_, or Co phosphate nanoparticles, which facilitate the extraction and utilization of the photogenerated charge carriers.

The conventional sample design to study PEC properties is powder‐based. For any potential future application, this will be the envisaged sample design mainly due to the relatively cheap device fabrication and the large surface area in contact with water where the OER takes place. However, using powder‐based samples, several fundamental materials’ properties are barely accessible since crystallinity, average grain size, crystallographic orientations of the grains, morphological features, and electrical properties are all interconnected and difficult to control with precision [9]. The use of pulsed laser deposition (PLD) to grow thin film model systems offers not only the possibility to select specific crystallographic orientations of the surface [9], but also has the advantage of providing samples whose PEC performance is independent of the sintering properties of the material. Thin film samples are fully dense, possess well‐defined surfaces and interfaces and the orientation can be selected. This is different for powders, where a different average grain size and the need to provide a good electrical contact (grain–grain; grain‐electrodes) can affect the PEC activity for reasons related to morphology and not only to intrinsic materials’ properties [9].

Concerning oxynitride semiconductors, the utilization of growth orientation control and facet engineering to enhance PEC properties has not yet been sufficiently explored, although significant difference in the photo‐charge extraction for different crystallographic surface orientation have been reported [2, 3]. This may be due to the existence of catalytically preferred surface facets or a surface reconstruction [7], or to anisotropic charge migration properties in the crystal. For instance, in the model system BiVO_4_, the reduction reaction with photogenerated electrons and oxidation reaction with photogenerated holes take place separately on (010) and (011) facets, respectively [10]. In BaTaO_2_N, the photocatalytic activity of samples with co‐exposed (100) and (011) facets is nearly 10‐times higher than that of BaTaO_2_N with only (100) facets [8, 11]. In LaTiO_2_N thin films we measured significantly different charge extraction properties (and PEC activity) using epitaxial thin films with different crystallographic orientations [9, 12]. Such findings highlight the importance of facet and orientation engineering to obtain more insights into the mechanisms of charge transfer, extraction, and electrochemical utilization.

**FIGURE 1:**
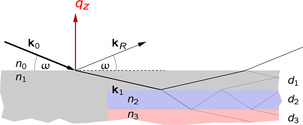
Schematic of the neutron reflectometry measurement on thin films. The incoming beam **k**_0_ is partially reflected on the surface of the sample (***k***_R_) and partly transmitted (***k***_1_). The same happens at the lower layer interfaces of the film, resulting in up‐ and down‐traveling waves in the layer system. All contributions leaving the sample into ***k***_R_ interfere, where the amplitudes and phases of the partial waves are functions of the densities (represented by the indices of refraction, *n*) and layer thicknesses *d*. The reflectivity curve *R*(*q*_z_) with *q*_z_ = 4π/λ sin ω is thus a function of the density depth (*z*) profile.

For the characterization of thin films heterostructures, reflectometry using X‐rays and neutrons is widely used [13, 14, 15]. These techniques use a highly collimated beam to probe flat surfaces and interfaces, and measure the intensity of the reflected radiation as a function of angle (for X‐rays) or angle and wavelength (for neutrons), as schematically shown in Figure 1. The reflectivity measurement provides information on the thickness and density profile in a single layer or heterostructures with a nanometric resolution. The neutron probe has unique features as compared to X‐rays, including a high contrast for light elements such as hydrogen, an isotopic sensitivity, and a large penetration depth into the bulk [16]. Using neutron reflectometry (NR), the scattering length density (SLD) depth profile can be deduced, which is a measure of the scattering power of the material. It increases with increasing the density of the material as well as the intrinsic scattering power of individual chemical elements. Thus, the SLD profile is directly related to the corresponding material composition distribution.

To obtain compositional and structural information, in particular for the light elements, we chose for this study to combine transmission electron microscopy (TEM) and neutron reflectometry (NR) as complementary techniques to characterize oxynitride thin films heterostructures.

For this study, SrTaO_2_N (STON) is selected as the photoactive oxynitride semiconductor to study the effects of the electrochemical activity at the solid/liquid interface. In the past, we have grown (001) and (011)‐oriented STON thin films to perform operando X‐ray absorption (XAS) spectroscopy during PEC characterization and compared the physicochemical evolution of STON thin films during OER with and without the presence of a thin NiO_*x*_ layer deposited in situ acting as co‐catalyst [17]. Here, to evaluate the effect of the PEC on STON with and without the catalytic NiO_*x*_ layer, TEM, electron energy loss spectroscopy (EELS), energy dispersive X‐ray (EDX) spectroscopy, and NR characterizations of STON epitaxial thin films with different crystallographic orientations are investigated and compared.

## 2 Results and Discussion

STON thin films with two crystallographic orientations ((001) and (011)) are fabricated using PLD on (100)‐oriented MgO and (0001)‐oriented Al_2_O_3_ substrates, respectively, as described in previous work [17]. A constant ammonia gas flow was used during the deposition, and the films were post‐annealed and cooled down in situ in the same gaseous environment.

As the current collectors for the PEC characterizations (001) and (011)‐oriented TiN films were grown on the MgO and Al_2_O_3_ substrates prior to the in situ growth of STON. Moreover, the TiN layers act as a nitrogen reservoir for the oxynitride layer growing on top and therefore help to increase the N content of the oxynitride film [17, 18].

As a co‐catalyst NiO_*x*_ was selected and deposited by PLD in situ onto the STON surface [19]. This layer was grown at a relatively low deposition temperature (*T*_S _= 300°C) to improve adhesion without promoting undesirable STON oxidation or interdiffusion of chemical elements at the interfaces. STON films with and without NiO_*x*_ co‐catalyst coatings were used for this study.

The supplementary schematic shows the sample designs, and Figures S2 and S3 show the XRD analysis of the two TiN‐STON‐NiO_*x*_ heterostructures grown on the two substrates. For the (001)‐oriented films a minor (011) diffraction peak was detected after the deposition of the co‐catalyst layer. We cannot exclude the presence of this secondary crystallographic orientation also for the samples as grown without NiO_*x*_. For the (011)‐oriented samples only this crystallographic orientation was detected.

The deposition rate of NiO_*x*_ was calibrated by XRR, as shown in Figure S4. The deposition rate and the compositional profile of the STON thin films was also calibrated using Rutherford back‐scattering as shown in Figure S5.

The standard 3‐electrode configuration is used for the PEC characterization. In this experimental setup, the MgO‐TiN‐STON(‐NiO_*x*_) photoanode acts as the counter electrode with the photoanode immersed into an aqueous electrolyte. The photo‐generated electrons are collected by the Pt wire contacted TiN layer acting as the working electrode. This is the location where the hydrogen evolution reaction takes place. The photo‐generated holes reach the NiO_*x*_/electrolyte interface and are consumed in the OER. A potentiostat is used to apply a voltage between working and counter electrodes with respect to a reversible hydrogen electrode (RHE). When the photoanode is illuminated, the PEC reaction starts and a current flows from the counter to the working electrode. This is referred to as the photocurrent and directly related to the amount of H_2_ and O_2_ generated by the photoelectrochemical system. Since a light‐induced current corresponds to the extracted charges, the measured photocurrent is an indication of the PEC performance. For the extraction of the photocurrent, linear sweep voltammetry (LSV) is performed by changing linearly the applied potential between 0.6 and 1.6 V versus RHE in one sweep and the current is normalized with respect to the light‐exposed film area. An intermittent illumination is used during the LSV measurements in order to separate voltage‐ and light‐induced effects.

**FIGURE 2:**
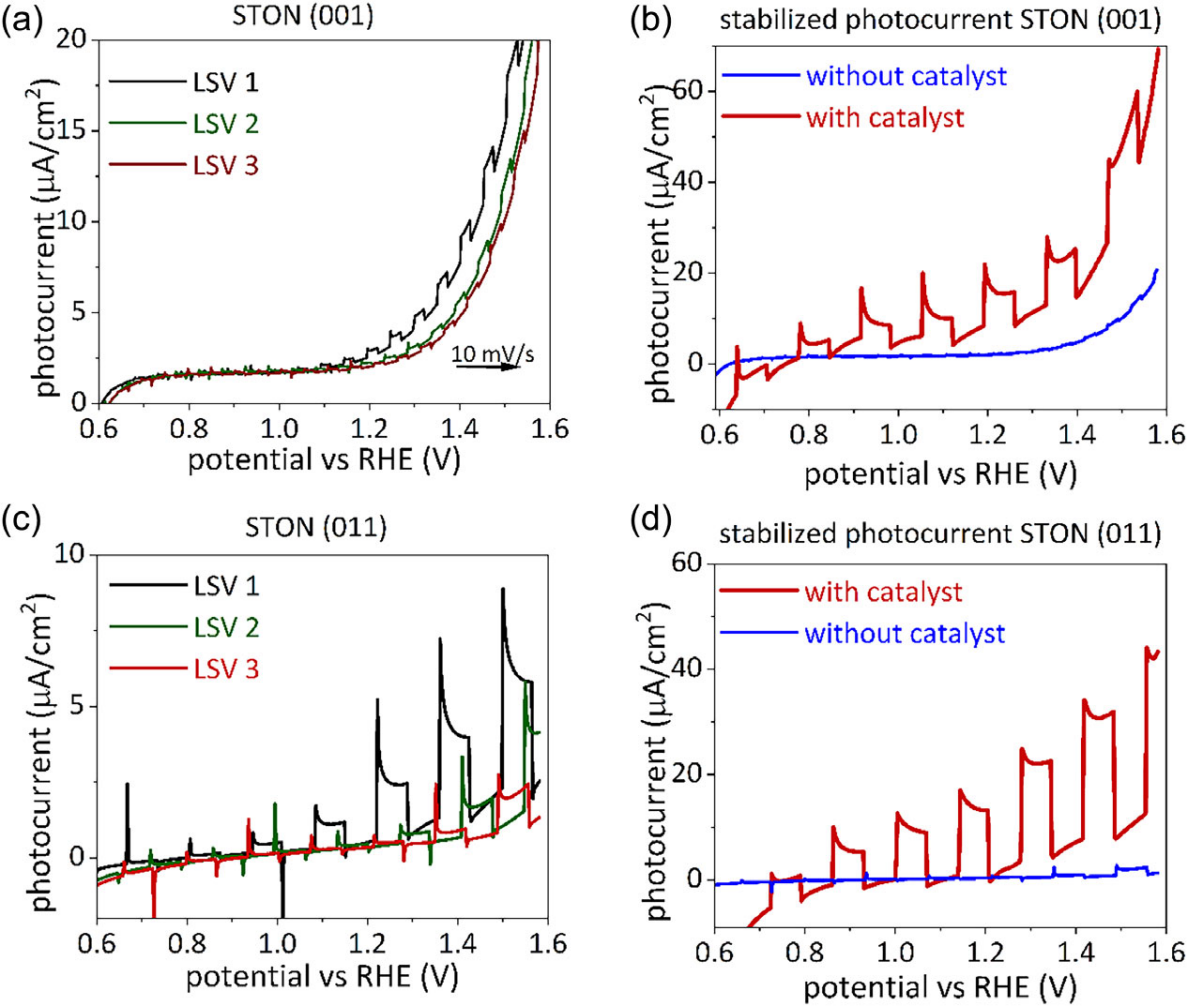
LSV scans of (a) bare STON (001) thin films under chopped light illumination, (b) third LSV scan STON (001) thin film under chopped light illumination with and without NiO_*x*_. LSV scans of (c) bare STON (011) thin films under chopped light illumination, (d) third LSV scan of STON (011) thin film under chopped light illumination with and without NiO_*x*_. The scans were taken at a scan rate of 10 mV/s from left to right.

Figure 2a shows the photocurrent of the bare STON (001) film photoanode under chopped light illumination. After the first LSV cycle, a 70% degradation in the photocurrent density can be observed for the next consecutive cycles. For a NiO_*x*_ coated STON (001) film the photocurrent remains stable after the LSV measurements with a minimal photocurrent degradation after completing three cycles (Figure 2b).

Figure 2c,d show the equivalent measurements for a STON (011) film with and without NiO_*x*_. Larger recombination peaks in the LSV scan can be observed for bare STON (011), compared to STON (001), as shown in Figure 2c. These peaks may indicate a larger initial charge recombination and hence a larger quantity of potentially available photocharges that are lost due to recombination [20].

The cocatalyst NiO_*x*_ (known to effectively transform into NiOOH at the surface under OER conditions) is rationalized for use from both practical and performance standpoints. This material was selected as the cocatalyst for STON among numerous possible alternatives, such as FeOOH, NiFeO_*x*_, NiCoO_*x*_, CoPi, and CoBi, because in a previous study we showed that NiO_*x*_ provides the best OER performance and the lowest onset potential when coupled with LaTiO_*x*_N_*y*_ oxynitride thin films [18, 20]. In addition, the deposition parameters for in situ NiO_*x*_ growth are compatible with those used for oxynitride deposition (in terms of available laser fluence, background pressure, temperature, and elemental composition). This compatibility enables in situ growth of the cocatalyst layer without exposing the sample to air, thereby minimizing potential contamination of the oxynitride/cocatalyst interface.

Like for STON (001) a similar 70% degradation in the photocurrent density is observed after the first LSV cycle (Figure 2c) and performance loss can be prevented adding the NiO_*x*_ co‐catalyst layer. In addition, the photoresponse with a NiO_*x*_ co‐catalyst for the (011) orientation is larger as compared for the (001) direction (Figure 2b–d).

A dependence of the photocurrent on the crystalline facet interfacing the electrolyte has also been observed for other oxynitride semiconductors, such as BaTaO_2_N [11, 20] and LaTiO_2_N [18, 20]. Such behavior may originate from variations in conductivity and charge recombination rates across different crystal planes and/or from differences in bulk‐to‐surface charge transport.

The photocurrent of STON (001) can be compared with a previous work in our group where values are practically equivalent (ca. 1 µA cm^−2^ at 1.3 V vs. RHE) [17]. Other bare thin film oxynitrides that have been tested without sacrificial reagents also possess very similar values such as LaTiO_*x*_N_*y*_ [9, 12, 20, 21], BaTaO_*x*_N_*y*_ [9], and Ta_3_N_5_ [22], where films have been fabricated via physical vapor deposition in order to obtain Nanometer‐scale smoothness. The addition of a cocatalyst can be difficult to compare considering there are many methods of its deposition; however, the enhancement is clear and has been seen to enhance our LaTiO_*x*_N_*y*_ thin films by 5–10 µA cm^−2^ [21]. Note that crystalline thin films have up to two orders of magnitude less surface area than a typical powder system, effectively decreasing the photocurrent density by as much.

The presence of an apparent cathodic currents at low potentials is attributed to the onset of the experiment (the beginning of the LSV), during which a small amount of capacitive charging is required to stabilize the sample, temporarily lowering the measured current. This effect is more pronounced for the “with catalyst” samples shown in Figure 2b,d, as the currents from these samples are larger in magnitude.

### 2.1 SrTaO_2_N Thin Films: Morphology and Composition

**FIGURE 3:**
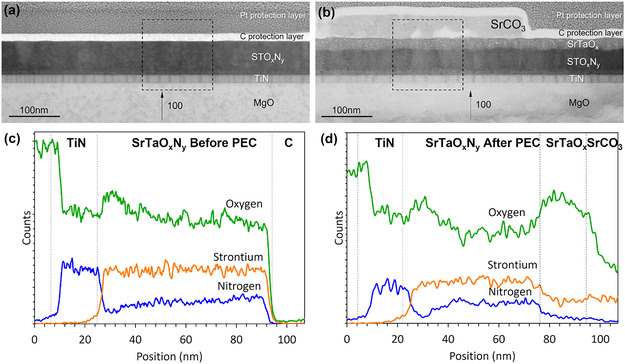
Low magnifications BF STEM images of STON (001)‐oriented films (a) before and (b) after PEC tests. (c) and (d): N, O, and Sr concentration profiles derived from the EDX data before and after PEC tests over corresponding selected areas marked in (a) and (b).

**FIGURE 4:**
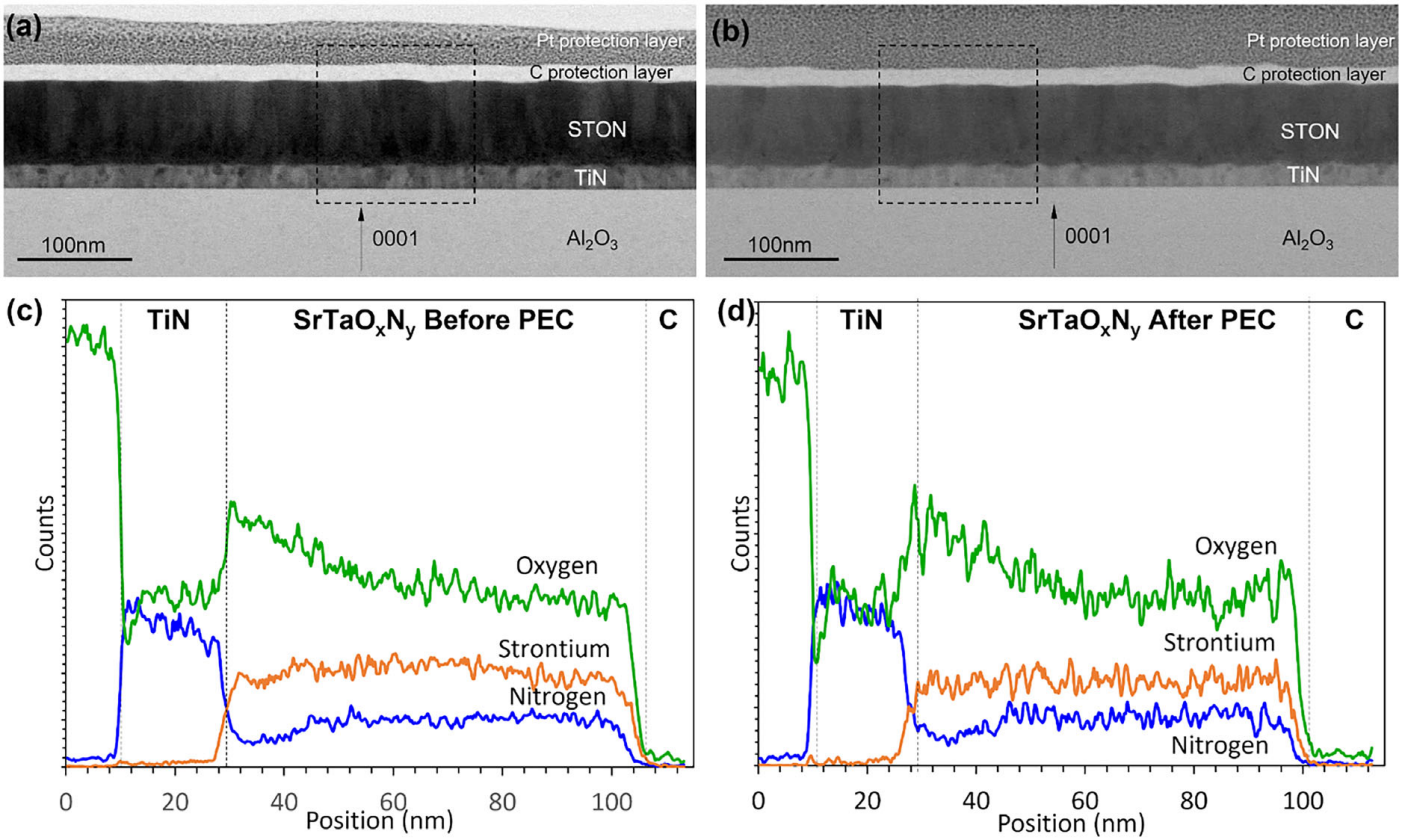
Low magnifications BF STEM images of STON (011)‐oriented films (a) before and (b) after PEC tests. (c) and (d): N, O, and Sr concentration profiles derived from the EDX data before and after PEC tests over corresponding selected areas marked in (a) and (b).

The PEC characterization of the different films used as photoanodes with and without co‐catalyst revealed clear differences in their photoresponse. To investigate the origin of these electrochemical differences, TEM, EELS, and EDX measurements were used for a detailed structural and compositional analysis. Figures 3 and 4 show the TEM and EDX analyses of the (001) and (011) oriented STON films before and after PEC tests.

For films grown on both substrates, cross‐section TEM and EDX of as‐grown samples show the typical features of uniform and well‐defined STON / TiN heterostructures (Figures 3a and 4a). The chemical composition profile, as revealed by EDX, is shown in Figures 3c and 4c for both samples.

The TiN layers, although grown along two different crystallographic orientations, show a very similar morphology with out‐of‐plane oriented columnar grains. EDX reveals grains of Fe to be the origin of such a morphological feature (Figure S6). As Fe is not present in SrTaO_*x*_N_*y*_ nor in the substrate, these Fe impurities are likely to originate from impurities in the TiN target. The inhomogeneous morphology of the TiN layer may cause the formation of defects at the interface of the SrTaO_*x*_N_*y*_ layer. As far as the functional role of this layer is concerned, i.e., as the current collector for PEC measurements, the presence of Fe impurity, although unwanted, is irrelevant. Figure S6 clearly show that Fe grains are embedded in the current collector and EELS show no evidence of Fe diffusion in the oxynitride layer.

As observed in previous studies, O is present in the TiN layer, which becomes TiO_*x*_N_*y*_ during the deposition of the oxynitride layer. The TiN layer can accommodate a relatively large O content without significantly affecting the crystal structure and the electronic conducting properties.

#### 2.1.1 SrTaO_2_N (001) – Comparison Before and After PEC Tests

Figure 3 shows TEM cross‐sectional images of the (001)‐epitaxially grown STON film before (a) and after (b) PEC. The C and Pt protection layers seen in the images were applied to the sample solely for the TEM measurements. Before PEC, the thickness of the STON film is uniform with well‐defined surface and interfaces between the layers (Figure 3a). EDX data (Figure S6) show that the cations (Sr, Ta in STON, and Ti in TiN) are uniformly distributed within their respective layers without evidence of significant interdiffusion.

After the PEC measurements, an amorphous and porous SrTaO_*x*_ layer appears on the film surface and SrCO_3_ islands are formed on top of the sample (Figure 3b, Figure S7). The SrCO_3_ layer is associated with the partial solubility of SrO in an alkaline electrolyte (pH = 14) to form ionic Sr^2+^ and 2OH^−^. Therefore, a dissolution and leaching of Sr^2+^ into the electrolyte takes place and a subsequent reaction with dissolved CO_2_ in the electrolyte. During OER, a dissolution of the A‐site cations occurs in perovskite structures [23, 24, 25]. This is of particular relevance for Sr, as SrO is soluble in water [26]. Many ABO_*x*_ perovskite structures tend to exhibit an AO surface termination [27, 28]. In the case of the STON thin film, the STON (001) oriented surface is expected to be SrO‐terminated [29]. Thus, Sr dissolution occurs spontaneously during OER.

The formation of an amorphous SrTaO_*x*_ surface layer points toward a loss of N as measured by TEM (Figure S7). Figure 3c,d show the distribution profile of Sr, O, and N before and after PEC, respectively. An increase in the O content and a local decrease in the N content can be seen at the interface between the TiN and the STOn layers. This effect has been observed in previous works and can be ascribed to O to N exchange at the interface. The formation of a Ti oxynitride layer at the interface does not affect either the crystalline structure or the metal‐like conducting properties of the current collector. After PEC, there is a strong decrease in the N:O ratios in the STON sample, with the layers close to the surface having almost no N (Figure 3d). Previous works report the amorphization of materials undergoing OER. This was observed for Ba_0.5_Sr_0.5_Co_0.8_Fe_0.2_O_3‐δ_ using TEM, a perovskite with high OER activities [30]. Instead, the literature reports contradictory results for La_0.4_Sr_0.6_CoO_3−δ_. It is reported to be stable during OER [31], whereas the formation of an amorphous surface layer was observed in [32].

For our samples, EDX also shows the presence of SrCO_3_ islands after PEC on the surface of STON in agreement with the hypothesis of Sr leaching with the subsequent formation of Sr‐containing oxides and carbonates at the surface, as confirmed by TEM (Figure 3b,d, and S7).

#### 2.1.2 SrTaO_2_N (011) – Comparison Before and After PEC Tests

The same TEM and EDX characterization experiments have been conducted for (011)‐oriented STON (Figure 4a,b). After the PEC characterization, there are no evident morphological changes for STON (011) in clear contrast to STON (001). The surface of the sample preserves its original structural features after PEC, showing crystallographic STON facets that are, at least morphologically, stable during OER.

However, also for this surface orientation EELS reveals major N loss in the near‐surface region where the N content after PEC is much lower than the original value (Figure S8). Figure 4c,d display the distribution profile of N, O and Sr throughout the film thickness before and after PEC measurements, respectively. As previously remarked, also for this crystallographic orientation we notice a decrease of the N content resulting in an oxygen increase in the interface layer between TiN and STON. Contrary to the STON (001) samples, we have no evidence of Sr leaching at the surface after PEC.

The (011) oriented samples do not undergo the dramatic physicochemical degradation observed for (001) facets. We conclude that the electrochemical stability during OER of (011) STON is much higher as compared to (001). However, N loss at the solid–liquid interface reduces the PEC performance of both samples as shown in Figure 2. This is in line with literature [17, 21] reporting that STON (011) exhibits both [011] and [021] orientations, which leads to a Ta‐ON surface termination [29]. Such a termination would create a B‐site cage [24] possibly inhibiting the dissolution of the A‐site cation (Sr), as well as the loss of O and N.

### 2.2 The Effects of NiO_*x*_ Surface Coatings

To characterize the effect of the NiO_*x*_ surface coating as an effective protecting layer to boost the electrochemical activity of the STON film, we grew in situ (001)‐ and (011)‐oriented STON films with a NiO_*x*_ layer about 40 nm thick. To improve the adhesion of the NiO_*x*_ a deposition temperature of 300°C was selected and the deposition rate calibrated by X‐ray reflectometry.

**FIGURE 5:**
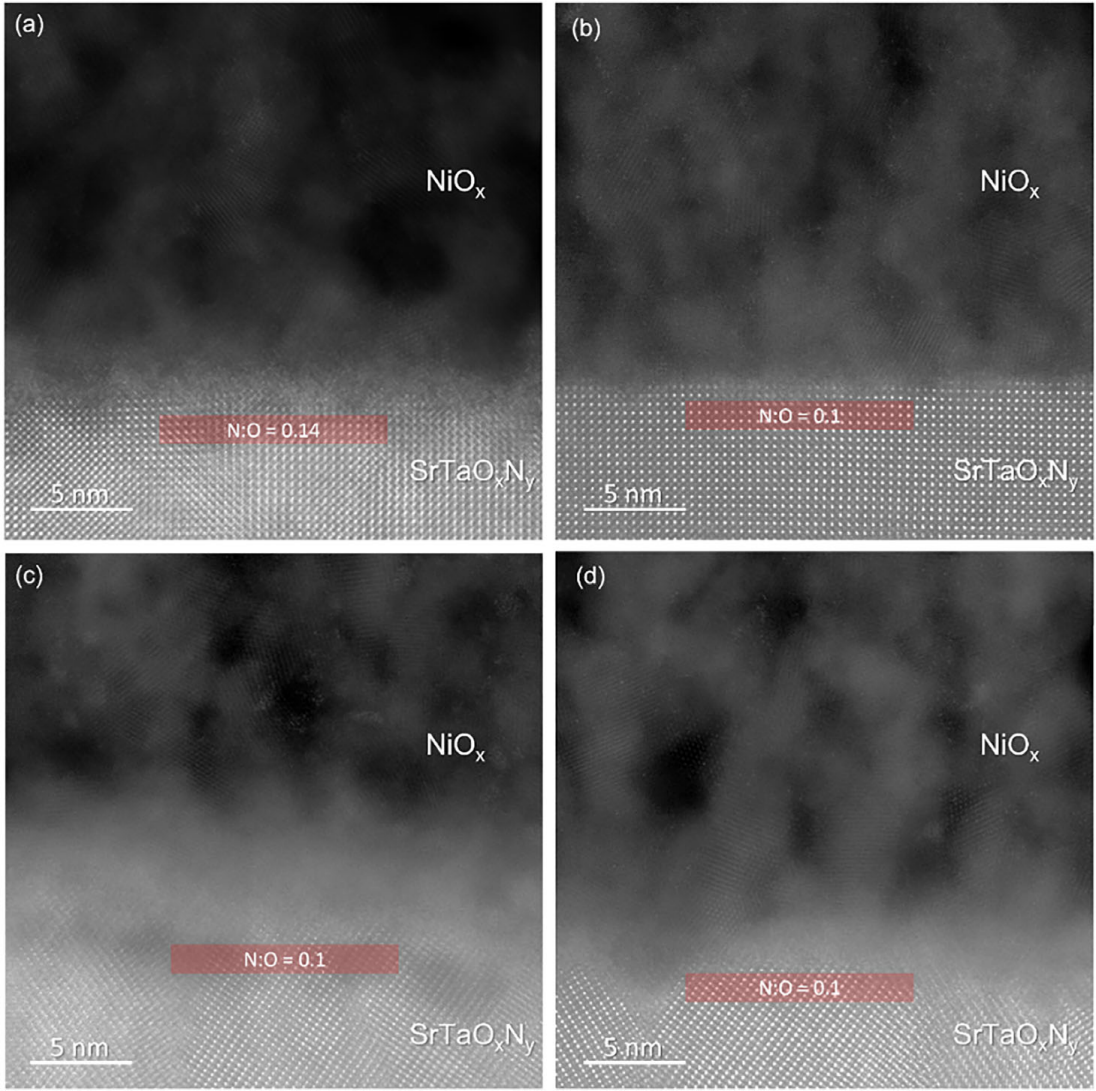
HR HAADF STEM images of NiO_*x*_/SrTaO_*x*_N_*y*_ interfaces for (001) (a) before and (b) after PEC measurements, and for (011) (c) before and (d) after PEC measurements, respectively.

Figure 5 shows high‐resolution HAADF images of STON/NiO_*x*_ interface for both STON orientations. The NiO_*x*_ layer in both cases consists of nanocrystalline grains with a lot of pores in between, showing an inhomogeneous morphology. For the NiO_*x*_‐coated samples, we did not observe any significant morphological change or a cation distribution before and after PEC. Both film orientations show slight Sr diffusion into the NiO_*x*_ layer (Figure S9) with no significant changes before and after PEC. We also observed the presence of an amorphous SrO_*x*_ interlayer between STON and NiO_*x*_. The formation of this interlayer is probably the result of exposing the STON surface to the O_2_‐containing atmosphere at 300°C during the in situ deposition of NiO_*x*_. The main finding of this EELS analysis is that after PEC the N:O ratio in the region near the STON/NiO_*x*_ interface remained unchanged for the (011)‐oriented STON sample, as shown in Figure 5c,d. For the (001)‐oriented STON sample instead, the N:O ratio decreased from 0.14 to 0.10 in the region near the interface with NiO_*x*_ (Figure 5a,b), whereas in the same region a complete *N* depletion was observed without NiO_*x*_, as shown in Figure S7b.

### 2.3 NR Characterization of SrTaO_2_N

Complementary NR measurements have been conducted on these STON samples to obtain information on their physicochemical changes as a result of the PEC process with and without NiO_*x*_ co‐catalyst. The compositional and structural information obtained from EDX, EELS, and TEM are used for a detailed modeling of the film density profile changes, which are related to the changes of their chemical compositions upon OER. The importance of this approach is that once the detailed mechanism of the OER effects is known, NR can provide a way to monitor the physicochemical changes of the sample in situ using an electrochemical cell reactor mounted directly at the neutron beamline.

In reflectometry, the beam hits the samples at a grazing angle and is partly transmitted and reflected. The interference of all partial beams from the surface and from all buried interfaces results in the reflected beam intensity. NR offers a depth resolution of a fraction of a Nanometer, it is non‐destructive and it can be applied to reveal buried interfaces, that are not easily accessible to other techniques. The neutron reflectivity is plotted as a function of the momentum transfer vector *Q*_z_ = (4π/λ) sinθ, where *λ* is the neutron wavelength and θ, the angle of incidence with respect to *z*, which is the direction of the substrate surface normal.

#### 2.3.1 SrTaO_2_N (001) NR Characterization With and Without Co‐Catalyst

**FIGURE 6:**
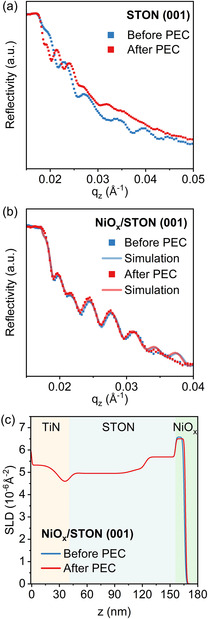
(a) Reflectivity plot for STON (001) film before and after PEC; (b) Reflectivity plot and corresponding model fit for STON (001) − NiO_*x*_ before and after PEC. The inferred SLD profile as a function of depth from the corresponding fit for (c) STON (001) − NiO_*x*_ before and after PEC.

Figure 6a shows reflectivity measurements before and after PEC for STON (001) samples without NiO_*x*_. There, a large difference is noted in the intensity, periodicity, and oscillation intensities reflecting the already discussed changes measured by TEM. Figure 6b shows the reflectivity measurement and data fit of STON (001) − NiO_*x*_ before and after PEC.

With the NiO_*x*_ coating, the observable changes in the NR measurements are minimal. Using the input from TEM measurements to model the reflectivity data in Figure 6b, compositional information is obtained, and the scattering length density (SLD) depth profiles are extracted (Figure 6c). Simulation of the STON (001) sample is not shown due to the extreme changes after PEC with the aforementioned formation of amorphous SrTaO_*x*_ and SrCO_3_ layers, in which NR would not provide additional insights. Such a severe chemical instability can lead to unpredictable density profiles depending on specific experimental conditions. This renders the modeling also in hindsight for a potential in situ approach less meaningful simply because the electrochemical system, bare (001) STON facets, are unstable under OER conditions.

#### 2.3.2 SrTaO_2_N (011) NR Characterization With and Without Co‐Catalyst

**FIGURE 7:**
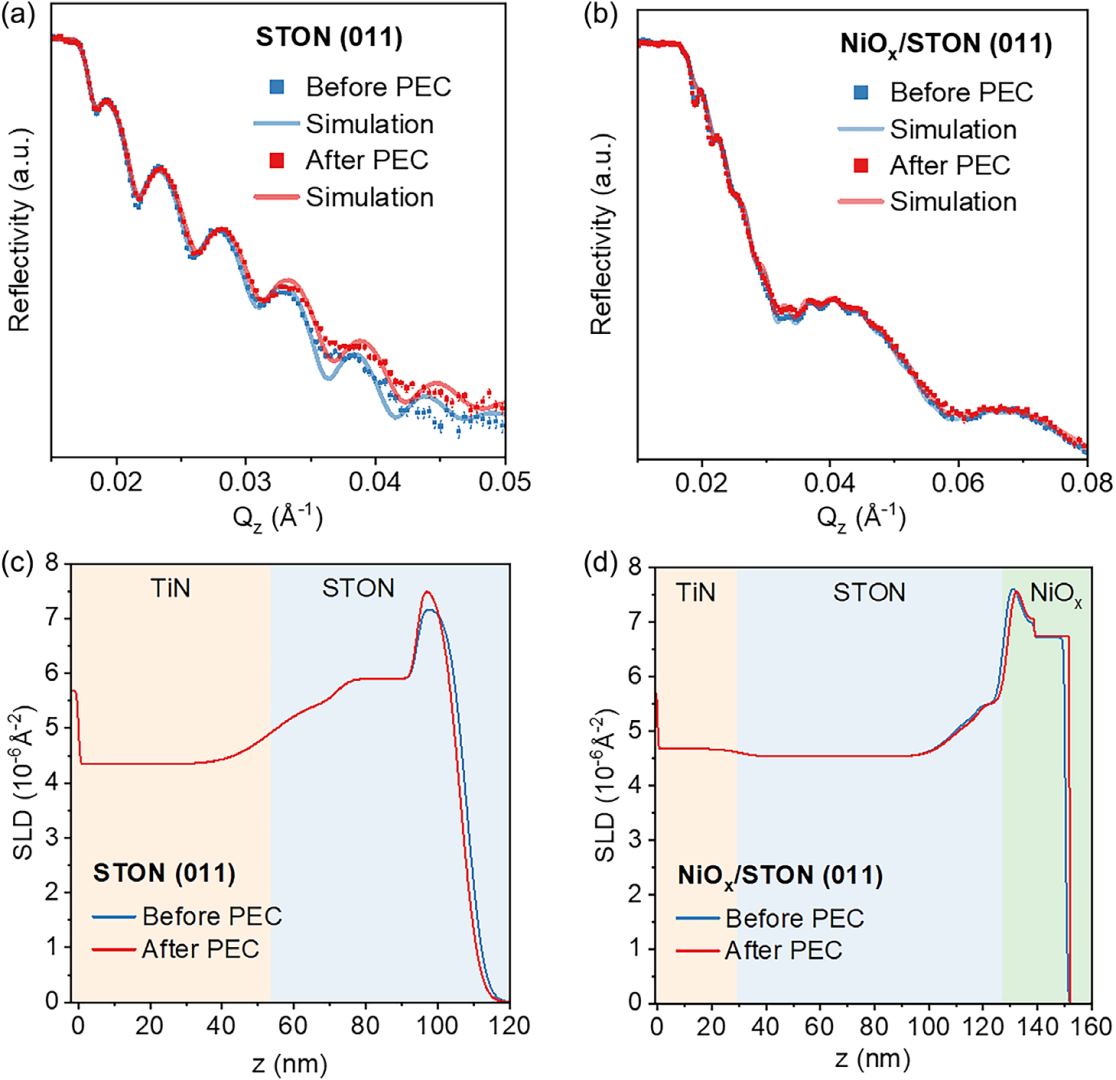
(a) Reflectivity plot and corresponding model fit for STON (011) film before and after PEC, (b) reflectivity plot and corresponding model fit for STON (011) − NiO_*x*_ film before and after PEC, (b) corresponding model fit for the data shown in (a). The inferred SLD profile as a function of depth from the corresponding fit for: (c) STON (011) before and after PEC. (d) STON (011) – NiO_*x*_ before and after PEC.

Modeling reflectometry data for (011)‐oriented STON with and without NiO_*x*_ by taking information obtained by TEM and EELS into account, a very good agreement between measured data and fit is reached as shown in Figure 7a,b. The (011)‐oriented films are electrochemically more stable during OER as compared to (001)‐oriented samples and NR measurements reflect this observation. This can be seen in Figure 7c,d, where the SLD profiles along the films do not show significant changes before and after PEC, except for a layer about 12 nm thick at the surface of the (011) STON without NiO_*x*_. This reflects the partial N depletion observed by EELS after PEC tests (Figure S8).

### 2.4 The Reconstruction of SrTaO_2_N Surfaces With (001) and (011) Facets

The NiO_*x*_ layer can form nickel hydroxide, Ni(OH)_2_, where Ni is in the +2 oxidation state. It is a stable compound in alkaline conditions in the pH range between 9 and 13 [33]. Further oxidation and deprotonation of Ni(OH)_2_ result in the OER‐active oxyhydroxide NiOOH compound. Several mechanisms for the OER reaction have been proposed for Ni‐based electrocatalysts [34]. Consequently, the uncertainties in the composition and structure of the active Ni‐based phases under OER conditions result in several different and controversial hypotheses on the origin of their OER activity. The O/H ratio and thus the Ni oxidation state can have several values [34]. For example, the full oxidation of Ni from +2 to +3 is as follows.

(1) $$\mathrm{Ni}{\left(\mathrm{OH}\right)}_{2}+{\mathrm{OH}}^{-}\to \text{NiOOH}+H_{2}O+e^{-}$$

As shown with TEM, the NiO_*x*_ layer comprises voids and is not homogeneous (Figure 5). Changes in the Ni oxidation, along with the adsorption of OH^‐^ radicals on the thin film, can change the NiO_*x*_ composition and thickness during PEC. The NiO_*x*_ layer hinders Sr leaching and N depletion upon PEC. However, the NiO_*x*_ layer also introduced changes at the interface with SrTaO_*x*_N_*y*_, such as a small Sr diffusion into NiO_*x*_ and a STON surface amorphization upon NiO_*x*_ deposition. The data obtained for the two out‐of‐plane crystallographic orientations investigated using TEM and NR are in full agreement with each other, implying that the observed changes can be measured using both techniques.

**FIGURE 8:**
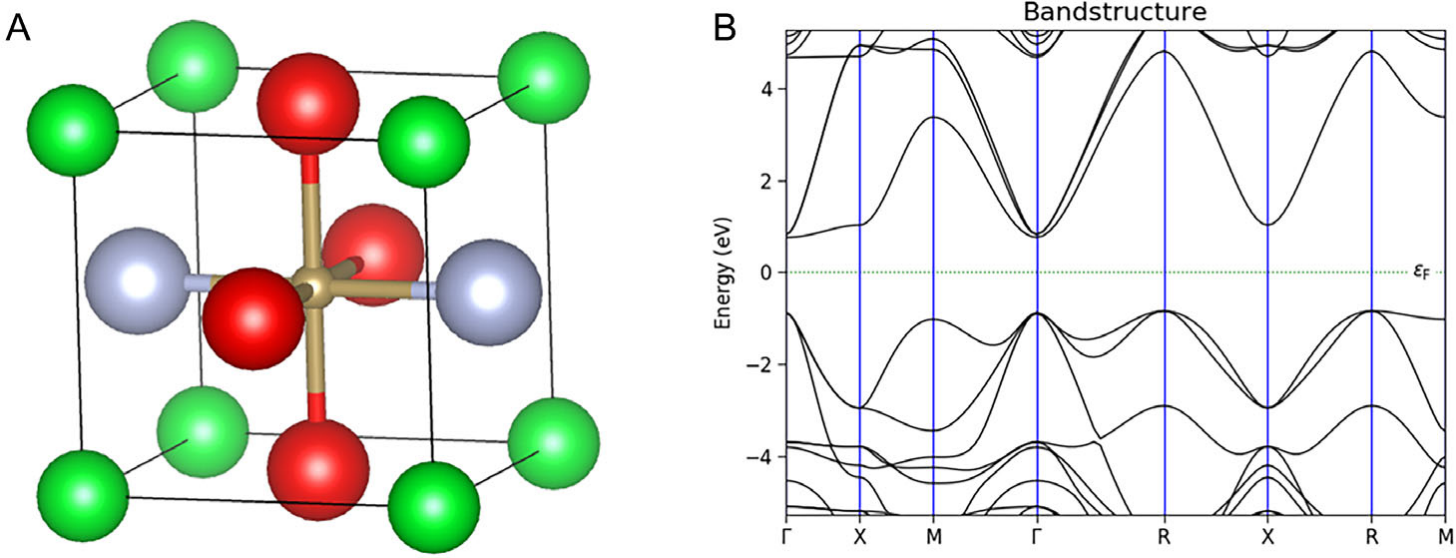
(A) Optimized cell and (B) band structure of SrTaO_2_N. Green color denotes Sr, brown Ta, red O, and gray N.

Density functional theory and Strongly Constrained and Appropriately Normed (SCAN) semi‐local Density Functional computations were used to rationalize the experimental findings. Figure 8 shows the optimized unit cell and band diagram of SrTaO_2_N with lattice parameters of *a* = *b* = *c* = 4.01 Å. The direct bandgap is calculated to be 1.63 and 1.59 eV for the indirect gap, which is smaller than the expected 2.1 eV, as the SCAN functional underestimates bandgap while providing accurate geometries and reaction energetics.

#### 2.4.1 The (001) Surface Termination

**FIGURE 9:**
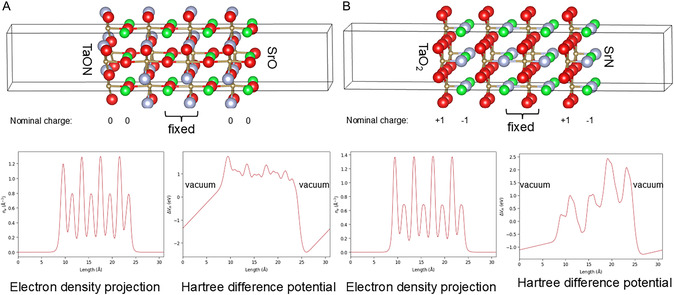
(A) SrO/TaON (001) terminated and (B) SrN/TaO_2_ 001 terminated SrTaO_2_N slabs with optimized geometries, nominal charges of the layers, electron density projections along the c direction of the slab and Hartree difference potential along the c‐direction of the slabs. Green denotes Sr, brown Ta, red O, and gray N.

Ionic surfaces obey the Tasker rules for surface dipole cancelations. According to those principles, the surface will undergo a reconstruction toward minimization of the surface dipole momentum [35]. The (001) surface‐slabs are characterized with SrO(N) termination on one side and TaON(O_2_) at the opposite side, as shown in Figure 9. According to the Tasker rules, the slab with a SrO/TaON termination should be a Tasker type 1 without surface dipole and the slab with a SrN/TaO_2_ termination should possess surface dipole, leading to severe surface reconstruction, a Tasker type 3 surface. The Tasker theory can be applied directly only to fully ionic materials where the actual electron density at the ions corresponds to their nominal charge. In complex oxides, due to metal–oxygen bond d‐p orbital hybridization and partial covalency, the actual electron density might differ significantly from the nominal charges. As a result, even surfaces classified as Tasker type 1 might exhibit surface dipole and severe reconstruction similar to Tasker type 3 surfaces.

Figure 9 summarizes the optimized geometries of 8‐layer slabs of SrO/TaON‐terminated and SrN/TaO_2_ (001) terminated SrTaO_2_N. The nominal charges are shown for both surface terminations. In addition, electron density projections along the *c*‐direction of the slab and Hartree difference potential along [001] are plotted. The Hartree difference potential plots reveal slops in the vacuum region for both terminations, which is a result of surface dipoles with opposite directions at both terminations of each slab. Asymmetric distribution of the electron density could be seen along the slabs.

Thus, both possible terminations behave as a Tasker type 3 surface and should undergo a reconstruction toward minimization of the dipole. While there is no general recipe for the direction of surface reconstruction, the general rule dictates that the surface layer should lose half of its charge. This is achieved more easily by removal of half of the atoms from one of the surfaces and moving them to the opposite surface. We select the AO‐terminated surface as it was shown as the stable (001) termination for most of the perovskites [27].

**FIGURE 10:**
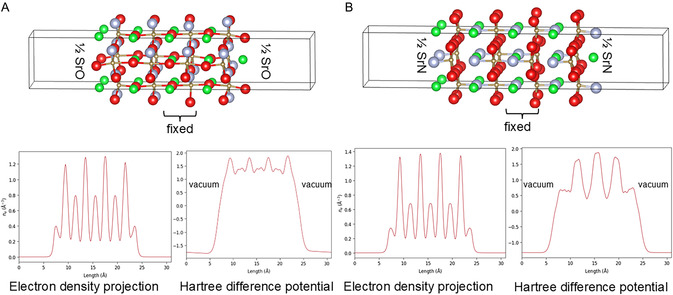
(A) Reconstructed 1/2 SrO (001)‐terminated and (B) 1/2 SrN (001)‐terminated SrTaO_2_N slabs with optimized geometries, electron density projections along the *c*‐direction of the slab and Hartree difference potential along the c direction of the slabs. Green denotes Sr, brown Ta, red O, and gray N.

Figure 10 summarizes the optimized geometries of reconstructed 8‐layer slabs of 1/2 SrO‐terminated and 1/2 SrN (001)‐terminated SrTaO_2_N. The electron density projections along the *c*‐direction of the slab and Hartree difference potential along [001] are plotted. The Hartree difference potential plots are flat in the vacuum region for both terminations, which shows that the surface reconstruction eliminated the surface vacuum. Symmetrical distribution of the electron density could be seen along the slabs.

The simple reconstruction of a 50% surface Sr loss and the equivalent atomic concentration of anion loss would lead to surface dipole elimination. The energy difference between the two slabs is below 70 meV, showing equal possibility for O and N loss.

#### 2.4.2 The (011) Surface Termination

The (011) surface termination consists of alternating layers of SrTaO/ON and SrTaN/2O. As a result, both cations are exposed on one side of the slab, and the other side is anion‐terminated. The nominal charge of the SrTaO layer is +5, the ON layer is −5, the SrTaN is +4, and the 2O is −4. In all cases, the slabs belong to the Tasker type 3 surface and should exhibit severe surface reconstruction.

**FIGURE 11:**
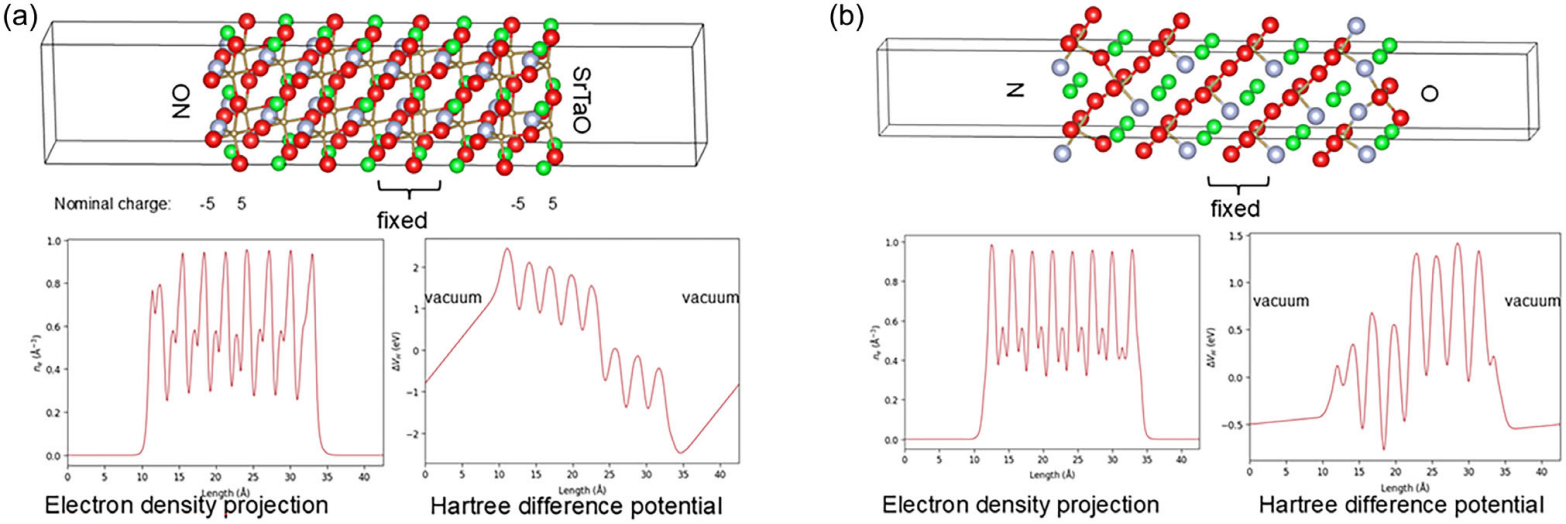
(a) SrTaO/ON (011)‐terminated SrTaO_2_N slab with optimized geometry, nominal charges of the layers, electron density projections along the *c*‐direction of the slab, and Hartree difference potential along the *c*‐direction of the slabs. (b) Reconstructed ½ O and ½ N (011)‐terminated SrTaO_2_N slab with an optimized geometry, electron density projections along the *c*‐direction, and Hartree difference potential along the *c*‐direction of the slabs. Green color denotes Sr, brown Ta, red O, and gray N.

The results for a SrTaO/ON (011)‐terminated SrTaO_2_N slab with an optimized geometry. Nominal charges of the layers, the electron density projections along the *c*‐direction, and the Hartree difference potential along the *c*‐direction of the slabs are summarized in Figure 11a. The slab exhibits a strong surface dipole and asymmetrical electron density distribution and hence will be subject to a surface reconstruction.

Different strategies to reconstruct the (011) surface are possible and our choice is halving the charge of the anion‐terminated surface on both sides, as it corresponds to a minimal reconstruction and minimal ionic displacement. Several possible structural configurations were explored, all of them yielding in slabs with a significant residual dipole momentum.

We have identified one possible structural modification that results in a dipole elimination, as shown in Figure 11b. The results for a reconstructed ½ O and ½ N (011)‐terminated SrTaO_2_N slab with an optimized geometry, electron density projections along the *c*‐direction and Hartree difference potential along [001] are summarized. In that slab, two surface terminations are possible: nitrogen‐enriched SrTa‐surface and O‐rich subsurface, or oxygen‐enriched SrTa‐surface and N‐rich subsurface. The dipole momentum of the ionic crystal has been neutralized by an ordered distribution of O and N in the surface/subsurface layers, opposed to the general slab dipole momentum. Such a cancelation was possible by the loss of 1/3 of the anions from the surface stoichiometry and ordered anion reconstruction. The dipole elimination was achieved without the necessity of cation loss within the top layers.

## 3 Conclusion

We have applied TEM and NR as complementary methods to investigate the effect of the photoelectrochemical oxygen evolution reaction at the surface of SrTaO_*x*_N_*y*_ thin films, a visible light‐responsive semiconductor used for the H_2_ evolution by solar water splitting. It is shown that the crystallographic orientation of the film plays a crucial role in the electrochemical stability of the surface during the oxygen evolution reaction. The STON (001)‐orientation displays significant Sr leaching and a loss of N. This leads to the formation of a SrCO_3_ and an amorphous SrTaO_*x*_ layer on the film surface. Such significant changes are not observed in the case of the STON (011)‐orientation. However, the (011) crystal facet also show a significant N depletion near the solid/liquid interface, leading to a dramatic reduction of the PEC performance.

The growth of a thin NiO_*x*_ layer on top of the SrTaO_*x*_N_*y*_ film significantly increases the electrochemical stability of the solid–liquid interface for both film orientations. By comparing before and after photoelectrochemical tests, the NiO_*x*_‐coated samples show only small changes of morphological, structural, and electrochemical properties.

Overall, the presence of Sr leads to leaching of the A‐site cation, and the SrTaO_*x*_N_*y*_ is not a stable structure. By selecting a suitable crystal facet and protection layer, these effects can be significantly reduced. Remarkably, the NR measurements match very well with the results obtained by TEM. The two experimental approaches complement each other. Therefore, taking advantage from the different cross‐section of neutrons with different chemical elements (and isotopes), NR to be used to study in situ the physicochemical evolution of the surface in contact with water, where the electrochemical reactions take place. This novel experimental approach may pave the way in future for the discovery and design of new materials and/or materials heterostructures with enhanced performance for solar hydrogen evolution.

Surface reconstructions and dipole compensating mechanisms were investigated for (001) and (011) facets of SrTaO_2_N slabs. We have shown that the pristine surface for both terminations exhibits a strong dipole momentum. We have proposed mechanisms for the dipole momentum compensations based on the guidelines of the Tasker theory for ionic surfaces. The (001) facet reconstructs to dipole‐free surfaces by the loss of half a SrO or SrN surface layer. Thus, this facet is prone to cation and anion loss through the formation of atomically disordered top layer. The (011) facet reconstructs through 1/3 of the surface anion loss to well‐ordered surfaces with an N‐excess in the top layer and an O‐excess in the subsurface layer, and vice‐versa. Such N/O ordering in the surface/sub‐surface layers generates opposite dipole momentum, which compensates the ionic surface dipole momentum.

## 4 Experimental Section/Methods

### 4.1 Thin Film Deposition

The oxynitride thin films presented in this work are grown using PLD. A KrF excimer laser (Lambda Physik LPX 300, 20 ns pulse width, *λ* = 248 nm) was used to ablate a polycrystalline Sr_2_Ta_2_O_7_ target. The SrTaO_*x*_N_*y*_ (STON) layer is grown at an N_2_ background partial pressure of 8.0 10^−4^ mbar, which was set via a gas inlet line to the vacuum chamber. In addition, NH_3_ gas is injected through a needle‐valve to reach a total partial pressure (N_2_ plus NH_3_) of about 5.0 10^−3^ mbar.

The laser was focused on a spot of 0.85 mm^2^ with a laser fluence of ca. 2.5 J cm^−2^. Commercially available (001) MgO and (0001) Al_2_O_3_ single crystal substrates are used (10 × 5 × 0.5 mm) and a target to substrate distance of 5 cm was chosen. Silver paste is applied between the substrates and the heating stage to provide good thermal contact. The substrate temperature was set at 750°C and was monitored with a pyrometer.

Before growing the STON films, the substrate was coated with epitaxial TiN layers used as the current collector for the photoelectrochemical tests. The TiN films were grown using a commercially purchased TiN target under vacuum with a base pressure of ca. 2 × 10^−6^ mbar. The substrate–target distance, substrate temperature, laser repetition rate, and fluence were the same as above.

With the intent of increasing the N content in the STON films, the STON‐TiN samples underwent an *ex situ* ammonolysis post‐treatment, with an NH_3_ flow rate of 200 mL min^−1^ at 750°C for 1 h. This ammonolysis step increased the N content by ca. 2% in the STON film. However, after ammonolysis the FWHM of the rocking curve measured by XRD at the STON XRD reflexes was almost twice the value as compared to as‐grown films, indicating that the ammonolysis step has detrimental consequences on the crystalline film quality. An optimization of the ammonolysis procedure to improve the crystalline quality was beyond the scope of this study. Based on our measurements, a potential trade‐off between crystalline quality and N content should be taken into consideration for planning future experiments.

The NiO_*x*_ deposition has been done on NH_3_‐postannealed STON samples and grown by PLD using a NiO_*x*_ target fabricated using a commercial NiO powder. Prior deposition, the PLD chamber is evacuated to a base pressure of 2 × 10^−6^ mbar to conduct the deposition in an oxygen background partial pressure of 0.45 mbar at a substrate temperature of 300°C monitored via a pyrometer.

### 4.2 Crystalline Properties

XRD measurements were performed using a BRUKER AXS D8 ADVANCE Bragg–Brentano Diffractometer with characteristic Cu K_α_ radiation 0.154 nm. θ−2θ scans were performed to determine the crystalline properties of the films.

### 4.3 Photoelectrochemical (PEC) Characterization

PEC measurements are performed using a three‐electrode configuration. The working and counter electrodes are the STON thin films and a platinum wire, respectively. A KCl saturated Ag/AgCl electrode is used as the reference electrode and an aqueous solution of 0.5 molar NaOH (pH = 14) is used as an electrolyte. For the electrical contact with the STON films, a wire is adhered to the TiN current collector to connect it to the potentiostat (Metrohm Autolab). The samples are irradiated for all experiments with a 150 W Xe arc lamp (Newport 66477) with an output intensity of 100 mW cm^−2^ calibrated with a photodetector (Gentec‐EO). To measure the dark and light current, the light intensity is modulated using a light chopper with a 30‐sec on–off cycle. Linear sweep voltammetry (LSV) measurements were performed at a scan rate of 10 mV/s under chopped illumination while varying the applied potential.

### 4.4 Chemical Composition Analysis by TEM

TEM specimens were prepared by focused ion beam lift‐out technique using a Thermofisher Scientific Helios (TFS, former FEI) UC instrument operated at 30 kV at the beginning of preparation, followed by 5 and 2 kV for the final cleaning. High‐angle annular dark field (HAADF), bright field (BF) images, as well as chemical maps, were collected with a probe‐side aberration‐corrected TFS Themis Z 80–300 TEM operated at 300 kV in scanning mode, equipped with a SuperX (TM) energy dispersive X‐ray (EDX) detector, and a Gatan Imaging Filter (GIF) Continuum 1065ER for Electron Energy Loss Spectroscopy (EELS).

### 4.5 Neutron Reflectometry (NR) Characterization

In conventional NR, either the angle or the wavelength is varied while the other is kept constant [36]. At the instrument Amor at PSI, a combined approach is used: The neutron wavelength is determined by using the time‐of‐flight from a chopper to the detector. And the focused beam of wide divergence (up to 1.5 deg, provided by a Selene optics is directed to the sample. The reflection angle is determined using a position‐sensitive detector [37, 38]. This approach allows for fast measurements of the specular reflectivity, but off‐specular contributions are not resolved and result in a higher background level [39]. Data analysis and modeling of the data were done using the GenX software [40].

### 4.6 Density Functional Theory

Calculations were performed using density functional theory and Strongly Constrained and Appropriately Normed (SCAN) Semilocal Density Functional [41, 42], as it is implemented in the QuantumWise ATK program [43]. We use pseudopotentials supplied by PseudoDojo library and medium‐sized basis set for all elements of numerical atomic orbitals. Spin polarization was not employed in the calculations. The simulations were performed for bulk cubic unit cell of SrTaO_2_N and slabs with (011) and (001) facet surfaces. Slabs included 8 layers of atoms and 2 nm of vacuum. The bandgap was estimated using the TB09LDA functional, known for its accurate, low computation cost performance for bandwidth estimation [44]. Surface polarization was approximated by Hartree difference potential plots and 1D‐electron density plots. The unit cell parameters were optimized until all forces and stress fell below 0.05 eV/Å using the LBFGS algorithm, and for the slab models, the forces on the atoms were relaxed until all forces below 0.05 eV/Å, while two atomic layers in the center of the slab were kept fixed.

## Supporting Information

Additional supporting information can be found online in the Supporting Information section. **Supporting** **Fig. S1**: Top: (100)‐oriented MgO single crystal substrate, (100)‐oriented TiN film, (001)‐oriented STON, NiOx layer. Bottom: (0001)‐oriented Al_2_O_3_ single crystal substrate, (111)‐oriented TiN film, (011)‐oriented STON, NiOx layer. **Supporting** **Fig. S2**: XRD plots of STON (001), (a) after NH_3_ annealing, (b) after NiO_x_ deposition and (c) after PEC. TiN and MgO have the same rock salt structure and similar lattice parameters (a=4.211Å (MgO), a=4.235Å (TiN)) with lattice mismatch of 0.56%. A TiN layer, therefore, grows in the (001) orientation on (001) MgO, and a (200) TiN diffraction peak is visible as a shoulder of the substrate Bragg peak. STON has a tetragonal perovskite structure with lattice parameters a=b=5.69080 Å, c=8.06860 Å. The XRD pattern shows that STON grows epitaxially on the TiN buffer layer with the (h,k,l) reflexes (002) and (004) appearing, although minor (110) out‐of‐plane oriented grains are detected after NiO_x_ deposition as well as after PEC tests. **Supporting** **Fig.** **S3**: XRD pattern of the epitaxially grown (011) STON film, (a) after NH_3_ annealing, (b) after NiO_x_ deposition and (c) after photoelectrochemical tests. **Supporting** **Fig.** **S4**: (a), θ–2θ scan and (b), XRR scans of NiO (200) grown on MgO (001) at different laser pulses. A growth rate of 2.05 nm/min is calculated for a laser fluence of 2.5 J/cm^2^. **Supporting** **Fig. S5**: RBS analysis of different STON thickness. **Supporting** **Fig. S6**: HAADF image of the (001)‐oriented STON film grown on TiN‐coated MgO substrate and corresponding chemical maps of C, Pt, N, Ti, Fe, O, Mg, Sr, and Ta. The columnar morphology of the TiN layer is clearly visible. **Supporting** **Fig. S7**: (a) HAADF image of the top layers cross‐section of the (001)‐oriented STON‐TiN film grown on MgO after PEC depicting SrCO_3_ and amorphous SrTaO_x_, (b) same TEM analysis showed in (a) at higher magnification. **Supporting** **Fig. S8**: (a) HAADF image of STON (011) after PEC, (b) N: O ratios measured by EELS at the surface and bulk of the STON (011) after PEC. **Supporting** **Fig. S9**: TEM cross section images and EELS elemental maps for Ni, Sr, and Ta for the NiO_x_‐STON (001) (top) and NiO_x_‐STON (011) (bottom). No significant changes were observed before and after PEC measurements.

## Funding

This study was supported by Schweizerischer Nationalfonds zur Förderung der Wissenschaftlichen Forschung (200020_204103), Brandenburger Staatsministerium für Wissenschaft, Forschung und Kultur, and Paul Scherrer Institut.

## Conflicts of Interest

The authors declare no conflicts of interest.

## Supporting information

Supplementary Material

## Data Availability

The data that support the findings of this study are available from the corresponding author upon reasonable request.
