# Supplementary material for: Characterization of Stable NiO x /SrTaO x N y Bilayers Boosting the Oxygen Evolution Reaction for Solar Water Splitting
Source: Small Sci. 2026 Mar 18;6(3):e202500638. doi: 10.1002/smsc.202500638 (PMC13098141; doi:10.1002/smsc.202500638)
Supplement: Supplementary file 1 — Supplementary Material [file SMSC-6-e202500638-s001.pdf]

Supporting Information

Characterization of stable $NiO_x/SrTaO_xN_y$ bilayers boosting the oxygen evolution reaction for solar water splitting

Zahra Pourmand Tehrani, Kyle J. Stephens, Vladimir Roddatis, Jochen Stahn, Aleksandar Staykov, Christof W. Schneider, Daniele Pergolesi, Thomas Lippert*

Zahra Pourmand Tehrani, Kyle J. Stephens, Jochen Stahn, Christof W. Schneider, Daniele Pergolesi*, Thomas Lippert
PSI Centre for Neutrons and Muons Sciences, Paul Scherrer Institute, 5232 Villigen PSI, Switzerland
E-mail: daniele.pergolesi@psi.ch

Zahra Pourmand Tehrani, Kyle J. Stephens, Thomas Lippert
ETH Zürich, Laboratory of Inorganic Chemistry, CH-8093 Zurich

Vladimir Roddatis
GFZ Helmholtz Centre for Geosciences, Telegrafenberg, 14473 Potsdam, Germany

Daniele Pergolesi

PSI Centre for Energy and Environmental Sciences, Paul Scherrer Institute, 5232 Villigen PSI, Switzerland

Aleksandar Staykov

International Institute for Carbon-Neutral Energy Research (I^2CNER), Kyushu University, 744 Motooka 819-0395 Fukuoka, Japan

((Please insert your Supporting Information text/figures here. Please note: Supporting Display items, should be referred to as Figure S1, Equation S2, etc., in the main text…)

Schematic of the samples

A schematic of the samples used in this study is shown in

Figure S1.

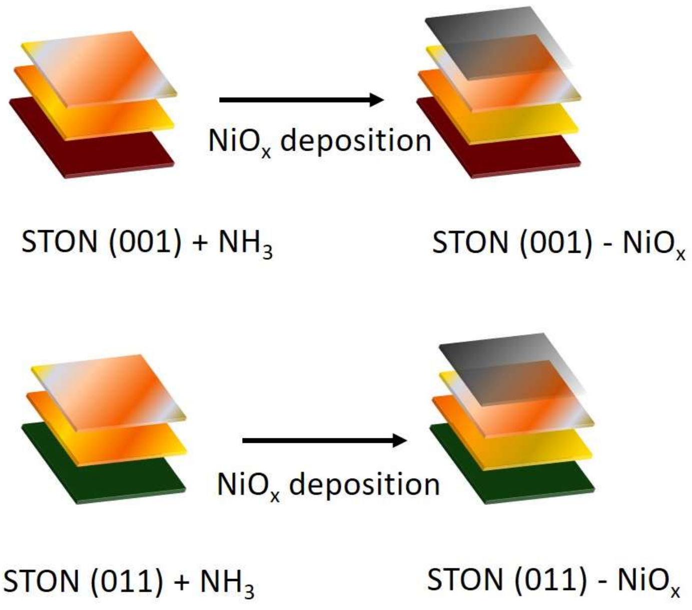

Figure S1: Top: (100)-oriented MgO single crystal substrate, (100)-oriented TiN film, (001)-oriented STON, NiOx layer.

Bottom: (0001)-oriented Al_2O_3 single crystal substrate, (111)-oriented TiN film, (011)-oriented STON, NiOx layer.

Crystallographic properties

STON (001)

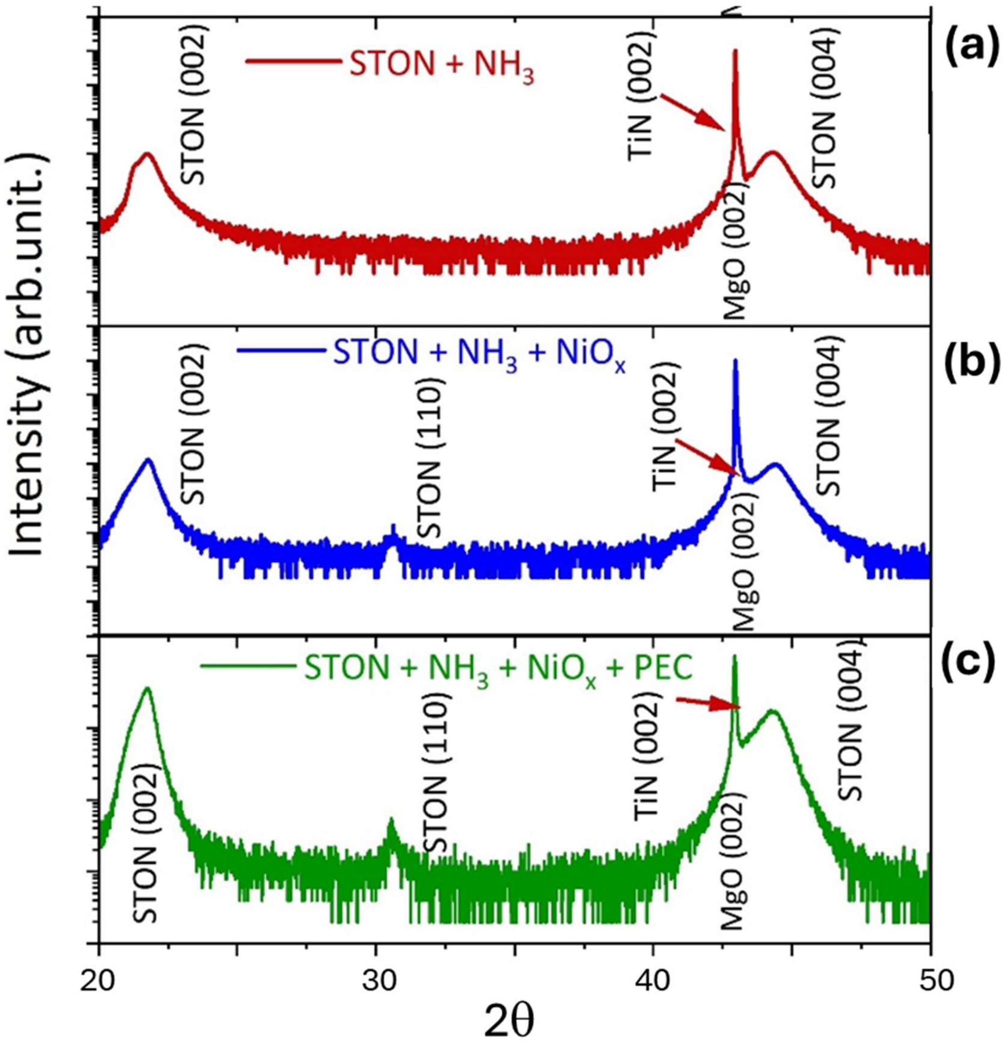

Figure S2: XRD plots of STON (001), (a) after NH_3 annealing, (b) after NiO_x deposition and (c) after PEC. TiN and MgO have the same rock salt structure and similar lattice parameters (a=4.211Å (MgO), a=4.235Å (TiN)) with lattice mismatch of 0.56%. A TiN layer, therefore, grows in the (001) orientation on (001) MgO, and a (200) TiN diffraction peak is visible as a shoulder of the substrate Bragg peak. STON has a tetragonal perovskite structure with lattice parameters a=b=5.69080 Å, c=8.06860 Å. The

XRD pattern shows that STON grows epitaxially on the TiN buffer layer with the (h,k,l) reflexes (002) and (004) appearing, although minor (110) out-of-plane oriented grains are detected after NiO_x deposition as well as after PEC tests.

STON (011)

Figure S3 shows the XRD plot of STON (011) after NH_3 annealing (red), after NiO_x deposition (blue) and after PEC reaction (green). For STON grown on Al_2O_3 substrates, TiN grows epitaxially along the (111) direction. The high quality of the TiN grown on Al_2O_3 is explained by the periodic arrangement of misfit dislocations that accommodate the large lattice mismatch (8.46%) at the film–substrate interface.

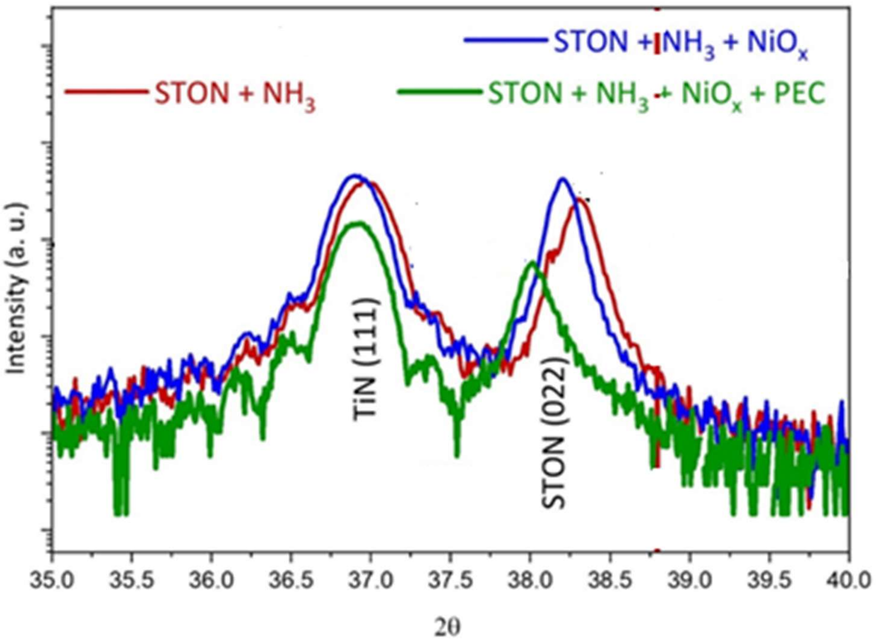

Figure S3: XRD pattern of the epitaxially grown (011) STON film, (a) after NH_3 annealing, (b) after NiO_x deposition and (c) after photoelectrochemical tests.

After the NiO_x deposition, the (022) peak shifts from 38.3081°to 38.2060°. Such a change in the 2θ position to lower values indicates changes in the size and shape of unit cells, possibly coming from the

loss of N and subsequent substitution with O. The peak position of TiN (111) also shifts to lower values suggesting that the O incorporation may also take place in the TiN layer. Previous observation showed slight diffusion of oxygen to the TiN layer from the oxynitride film [1] . Although TiN is thermodynamically stable, oxidation can take place in this material when exposed to oxygen, and the formation of TiO_2 and TiO_xN_y has been previously reported [2][3]. After the PEC reaction, the (022) peak further shifts to lower 2θ values, and a decrease in peak intensity is observed, suggesting N loss and changes in the $SrTaO_xN_y$ lattice.

NiO_x coating

The protective NiO catalytic layer is deposited based on previous work at 350°C [4]. MgO is a suitable substrate to investigate the epitaxial growth of NiO due to the common rock-salt crystal structure with similar lattice constants (MgO: 0.4212 nm); (NiO: 0.4176 nm) and low lattice mismatch < 1% [5]. The out-of-plane orientation of layers grown on MgO (100) at a different number of pulses is investigated by XRD shown in **Figure S4 (a)**. The NiO (200) and the MgO (200) substrate peaks are present, indicating exclusively (100)–oriented films. Next to the NiO (200) peak, Laue oscillations are observed indicating the growth of very smooth films [5]. X-ray reflectometry (XRR) is used to measure the thickness and growth rate of the NiO (200) on MgO, as shown in **Figure S4 (b)**.

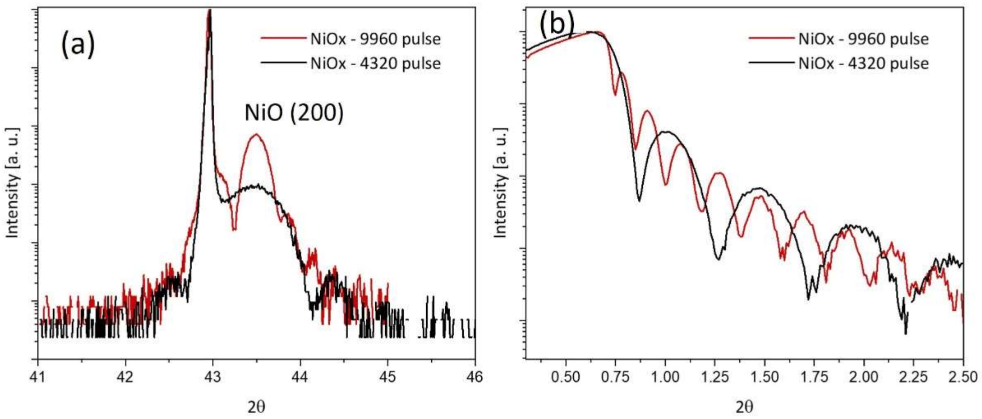

Figure S4: (a), θ–2θ scan and (b), XRR scans of NiO (200) grown on MgO (001) at different laser pulses. A growth rate of 2.05 nm/min is calculated for a laser fluence of 2.5 J/cm^2.

Thickness calibration of STON thin film using Rutherford Back Scattering (RBS)

Thickness calibration of the $SrTaO_xN_y$ layer is done using RBS data of films prepared with a different number of pulses (see

Figure S5). The composition profile of Sr and Ta demonstrate the homogeneous distribution of both elements. However, in the case of higher pulse numbers (10500 and 12600 pulses), the Ta profile is no longer homogeneous as verified using cross-section TEM. To employ films with a potentially homogeneous elemental distribution, 8400 pulses corresponding to an approximately 94 nm film thickness have been used for all fabricated samples.

RBS spectrum	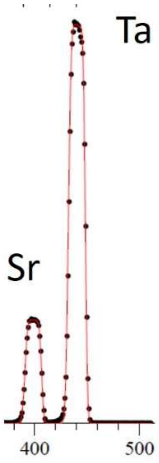	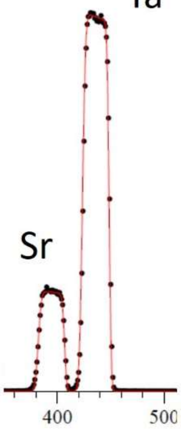	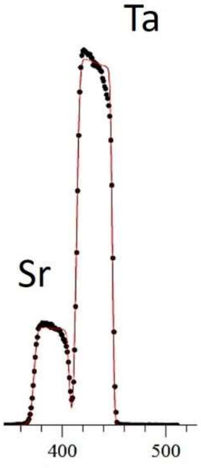	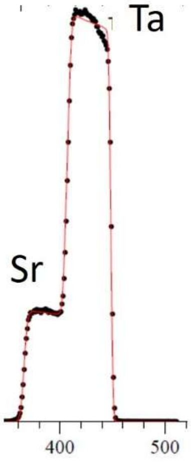
Number of pulses	4200	8400	10500	12600
Thickness (nm)	57.5	94.14	130.75	173.25

Figure S5: RBS analysis of different STON thickness.

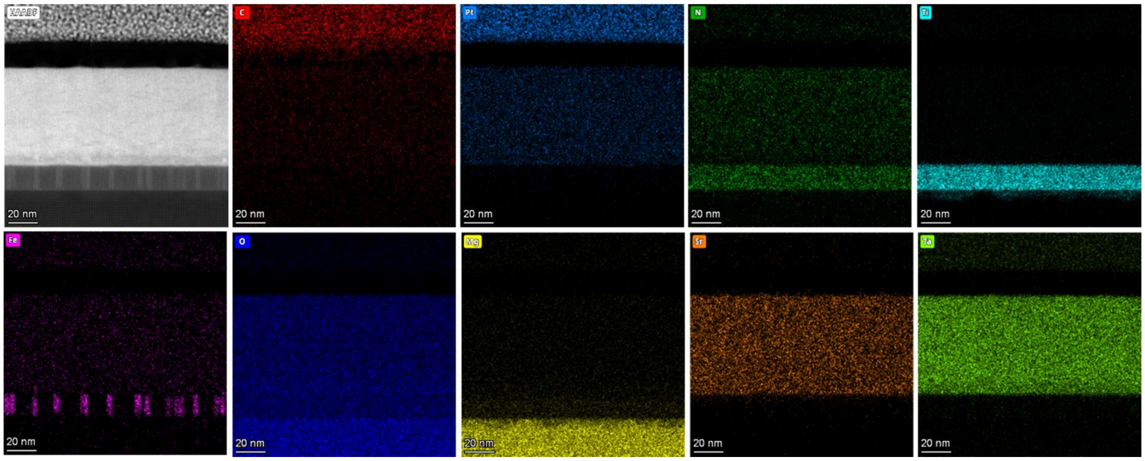

Figure S6: HAADF image of the (001)-oriented STON film grown on TiN-coated MgO substrate and corresponding chemical maps of C, Pt, N, Ti, Fe, O, Mg, Sr, and Ta. The columnar morphology of the TiN layer is clearly visible.

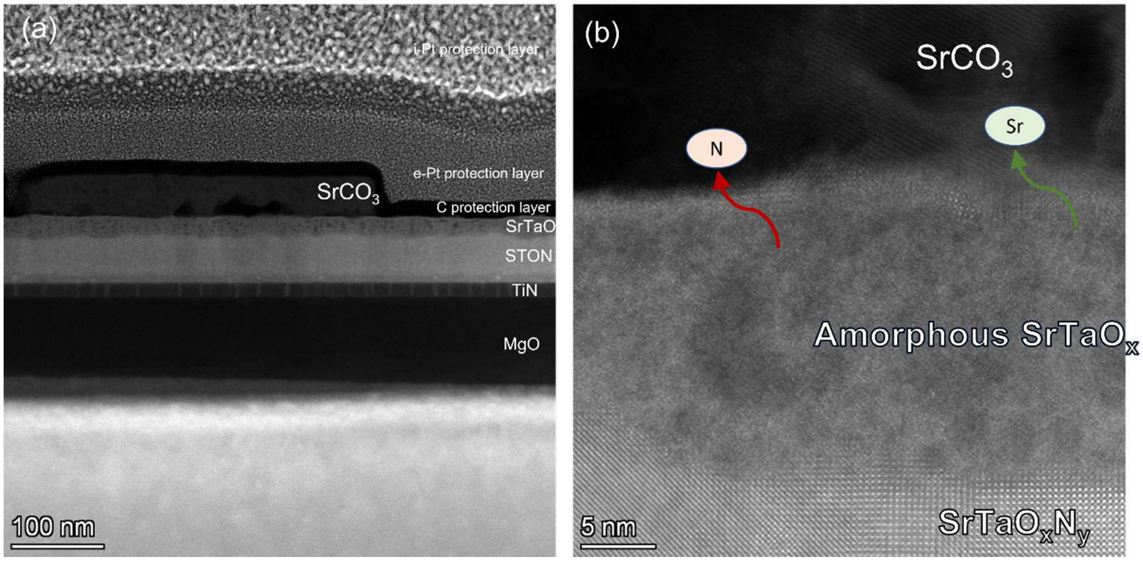

Figure S7: (a) HAADF image of the top layers cross-section of the (001)-oriented STON-TiN film grown on MgO after PEC depicting $SrCO_3$ and amorphous $SrTaO_x$, (b) same TEM analysis showed in (a) at higher magnification.

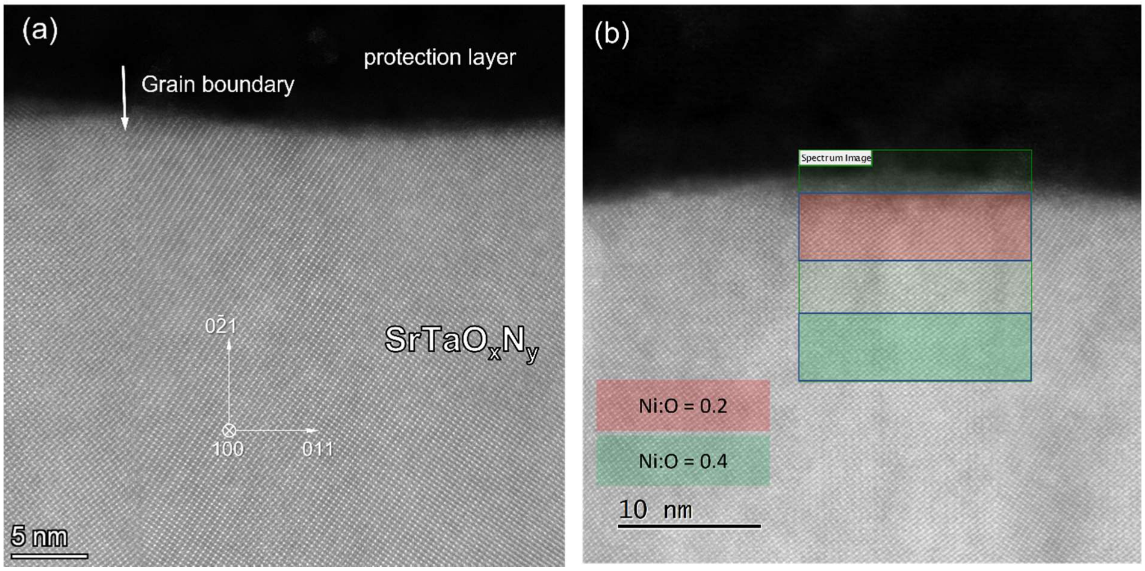

Figure S8: (a) HAADF image of STON (011) after PEC, (b) N: O ratios measured by EELS at the surface and bulk of the STON (011) after PEC.

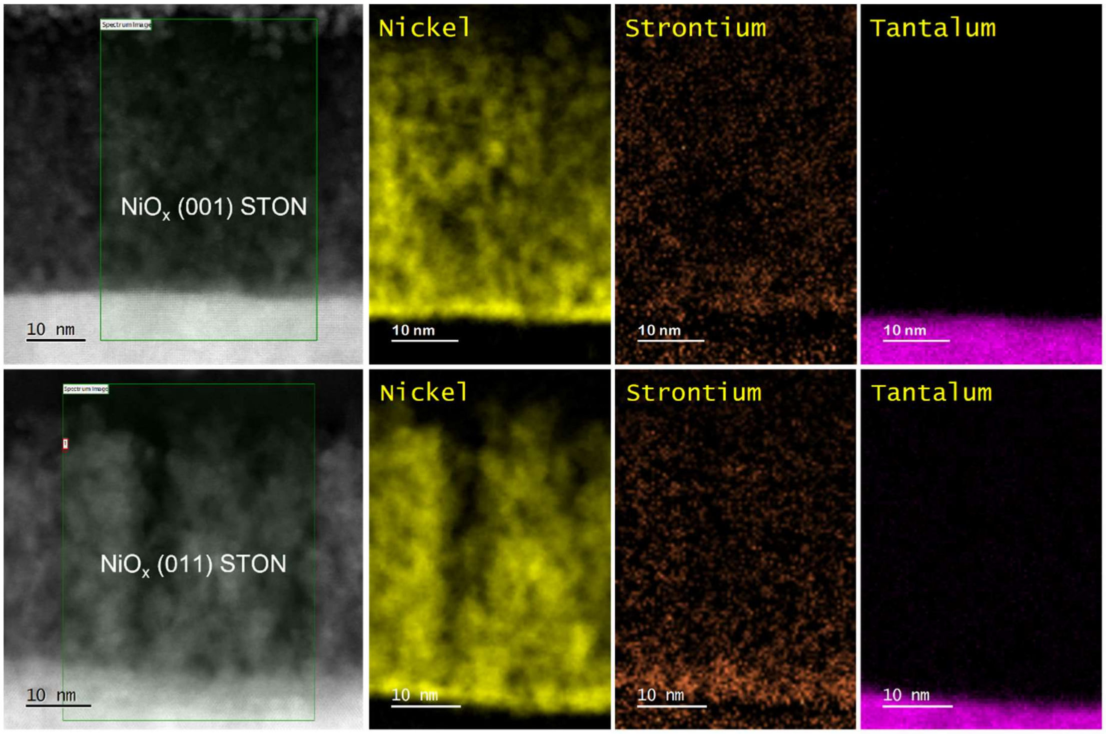

Figure S9: TEM cross section images and EELS elemental maps for Ni, Sr, and Ta for the NiO_x-STON (001) (top) and NiO_x-STON (011) (bottom). No significant changes were observed before and after PEC measurements.

Supplementary References

[1] C. Lawley *et al.*, "Examining the surface evolution of $LaTiO_xN_y$ an oxynitride solar water splitting photocatalyst," *Nat. Commun.*, vol. 11, no. 1, pp. 1–11, 2020.

[2] I. Montero, C. Jiménez, and J. Perrière, "Surface oxidation of TiN_x films," *Surf. Sci.*, vol. 251–252, no. C, pp. 1038–1043, 1991.

[3] H. Z. Wu, T. C. Chou, A. Mishra, D. R. Anderson, J. K. Lampert, and S. C. Gujrathi, "Characterization of titanium nitride thin films," *Thin Solid Films*, vol. 191, no. 1, pp. 55–67, 1990.

[4] A. Mazzi *et al.*, "Pulsed laser deposition of nickel oxide films with improved optical properties to functionalize solar light absorbing photoanodes and very low overpotential for water oxidation catalysis," *Mater. Sci. Semicond. Process.*, vol. 97, no. February, pp. 29–34, 2019.

[5] M. Budde *et al.*, "Structural, optical, and electrical properties of unintentionally doped NiO layers grown on MgO by plasma-assisted molecular beam epitaxy," *J. Appl. Phys.*, vol. 123, no. 19, 2018.

[6] B. J. Kirby *et al.*, "Phase-sensitive specular neutron reflectometry for imaging the nanometer scale composition depth profile of thin-film materials," *Curr. Opin. Colloid Interface Sci.*, vol. 17, no. 1, pp. 44–53, 2012.
